# Polyphenol-rich extract of *Ocimum gratissimum* leaves prevented toxic effects of cyclophosphamide on the kidney function of Wistar rats

**DOI:** 10.1186/s12906-021-03447-3

**Published:** 2021-11-02

**Authors:** Quadri K. Alabi, Rufus O. Akomolafe, Joseph G. Omole, Ayodeji Aturamu, Mokolade S. Ige, Oyindasola O. Kayode, Deborah Kajewole-Alabi

**Affiliations:** 1 Department of Physiology, Faculty of Basic Medical Sciences, Adeleke University, Ede, Osun State Nigeria; 2 Department of Physiological Sciences, Faculty of Basic Medical Sciences, Obafemi Awolowo University, Ile-Ife, Osun State Nigeria; 3 Department of Physiology, Faculty of Basic Medical Sciences, Afe Babalola University, Ado-Ekiti, Ekiti State Nigeria; 4 Department of Anatomy and Cell Biology, Faculty of Basic Medical Sciences, Obafemi Awolowo University, Ile-Ife, Osun State Nigeria; 5 Department of Public Health, Faculty of Basic Medical Sciences, Adeleke University, Ede, Osun State Nigeria; 6 Department of Biochemistry, Faculty of Basic Medical Sciences, University of Medical Sciences, Ondo, Ondo State Nigeria

**Keywords:** Antioxidant, Creatinine, Cyclophosphamid, *Ocimum gratissimum* leaf, Kidney, Sodium

## Abstract

**Background:**

Cyclophosphamide (CP) is one of the potent and low cost chemotherapy used in clinical setting against a variety of tumors. However, its association with nephrotoxicity limits its therapeutic use. *Ocimum gratissimum* leaf is a medicinal plant with numerous pharmacological and therapeutic efficacies, such as antioxidant, anti-inflammation, and anti-apoptotic properties.

**Methods:**

The present study was designed to evaluate the protective effect of *Ocimum gratissimum* (OG) against CP-induced kidney dysfunction in rats. Rats were pre-treated with 400 mg/kg b.w. of leave extract of *Ocimum gratissimum* (*Ocimum G.*) for 4 days and then 50 mg/kg b.w. of CP was co-administered from day 5 to day 7 along with *Ocimum G.* Markers of renal function and oxidative stress, food and water intake, electrolytes, aldosterone, leukocytes infiltration, inflammation and histopathological alteration were evaluated.

**Results:**

Obvious renal inflammation and kidney injuries were observed in CP treated groups. However, administration of leave extract of *Ocimum G.* prevented oxidative stress, kidney injuries, attenuated inflammation, increased aldosterone production and reduced sodium ion and water loss in rats. The plasma creatinine, urea and urine albumin concentration were normalized after the administration of *Ocimum G.* extract in rats treated with CP. *Ocimum G.* also decreased the plasma concentrations of Interleukin-(IL)-6, C-reactive protein and activity of myeloperoxidase and malondialdehyde in CP treated rats.

**Conclusion:**

*Ocimum G.* prevented kidney injury and enhanced renal function via inhibiting inflammation and oxidant-induced CP toxicity. The efficacy of *Ocimum G.* is related to the presence of various phytochemicals in the plant.

## Background

Cyclophosphamide (CP) is an antineoplastic compound that is associated with nitrogen mustard. It is used in the clinical setting to treat a wide variety of cancer diseases and in immunosuppressive therapy after organ transplants, treatment for autoimmune disorders such as rheumatoid arthritis, Wegener’s granulomatosis, and nephritic syndrome in children [1]. Regardless of the wide spectrum of clinical uses of the drug, CP is known to cause numerous dose-dependent organ injuries, which, therefore, restraining its therapeutic use in the clinical setting. As it was known to be beneficial in the treatment of nephritic syndrome in children [1], it was conversely reported in some previous studies to induce urinary bladder damage [2, 3] and cause renal toxicity when used inappropriately owing to the use of CP overdose [4–6].

The CP active metabolite, 4-Hydroxycyclophosphamide, is partially tautomerized into aldosphosphamide, which in turn is broken down by phosphatase in the circulation and live cell and give rise to two cytotoxic metabolites, phosphoramide mustard and acrolein [6]. The therapeutic action of phosphoramide mustard has been previously reported to be responsible for anti-tumor effects [6], while acrolein is responsible for cytotoxic effects of CP, such as hemorrhagic cystitis during the drug administration [2, 7]. The CP toxic metabolite damages the living tissues by abating the tissue endogenous antioxidant system through the generation of reactive oxygen free radicals which are mutagenic to living cells.

Administration of high dose of CP has been reported to increase lipid peroxidation and causes reduction in the endogenous enzymatic and non-enzymatic antioxidants [7, 8]. Previous studies have described the beneficial uses of antioxidants substances along with chemotherapy in preventing the drugs side effect on the body tissues [7, 9, 10]. The antioxidant agent like kolaviron proved effective in the prevention of CP cardiotoxicity in rats [11]. Thus, the combination of the antioxidant agent along with chemotherapy drugs may be a potential therapeutic approach to prevent or stop the cytotoxic development of CP.

*Ocimum gratissimum* (*Ocimum G.*) is a family of Lamiaceae which belong to herbaceous plant species. It is known as clove or African basil in many tropical areas countries (Asia, India, Brazil and West Africa etc). It is vastly found in Nigeria [12]. Many states of this country (Nigeria) used *Ocimum G.* as a sauce and vegetable soup. The leaves and flowers of *Ocimum G.* have been found to possess polyphenols and other bioactive compounds [13, 14]. *Ocimum gratissimum* plants are used to treat various illnesses like diarrhea, ulcerative colitis, fever, and catarrh [14–17]. The plant has many pharmacological advantages due to its anti-inflammatory, anti-oxidative, antibacterial, antidiabetic and antimalarial capabilities [14, 18–20]. The anti-microbial activity of the *Ocimum G.* extract has been tested in vitro against various human pathogens like *Klebsiella pneumoniae, Staphylococcus aureus, Escherichia, Vibro spp, Enterobacter spp, Enterococcus faecalis,* etc. The antifungal activity of the essential oil of *Ocimum G.* extracts has been noted in vitro [19, 21, 22], and *Ocimum G.* has been used as a mosquito appellant [23]. A recent animal study suggested the importance or protective effect of *Ocimum G.* extracts in the prevention of blood pressure irregularities against cobalt-chloride toxicity [24] as well as attenuating cardiac abnormalities associated with liver fibrosis by downregulating the interleukin-6 signaling pathway [25] or by inducing antioxidant effects [26]. Aqueous leave extract of *Ocimum G.* leaves showed antioxidant effect against cobalt chloride-induced cardio-renal injury [24] and prevented gastric ulcers by reducing gastric acid secretion and ulceration [27]. Also, urosolic acid from *Ocimum G.* extracts demonstrated anti-sickling activity [28] and anticancer [29, 30]. Furthermore, the essential oils of *Ocimum G.* have been shown to exhibit endothelium-dependent, vasorelaxant properties in rats [31]. In vivo and in vitro studies have evaluated eugenol, a medicinal constituent of *Ocimum G.*, to lower blood glucose levels via inhibition of alpha-glucosidase [32]. More so, a clinical study established the pharmacological adequacy of *Ocimum G.* to exhibit similar activity as chlorhexidine against plaque and gingivitis [33]. With all these features of *Ocimum G.*, we hypothesized that *Ocimum G.* might be a possible therapeutic when use along with CP to prevent or stop CP adverse effect. This study was therefore designed to examine the beneficial effects of polyphenol rich extract of *Ocimum gratissimum* leaves on CP-induced nephrotoxicity in male Wistar rats.

## Materials and methods

### Drugs, chemicals and instruments

Cyclophosphamide (200 mg/10 mL) injection was procured from Celon Laboratory (Hyderabad, India); ketamine hydrochloride (50 mg/10 mL) injection was purchased from Popular Pharmaceuticals Ltd., (Gazipur, Bangladesh); Assay kits for biochemical parameters were obtained from Randox Laboratories Limited, (Crumlin, UK) etc. 1-diphenyl-2-picrylhydrazyl (DPPH), methanol, sodium carbonate, trolox, and Folin–Ciocâlteu reagent were purchased from Merck & Co., Kenilworth, NJ, USA. The instruments used include the gas chromatography-mass spectrometer (GCMS-QP2010 SE, Shimadzu Corporation, Kyoto, Japan), an ultraviolet–visible spectrophotometer (Beckman, DU 7400, USA), Soxhlet apparatus, rotary evaporator, microscope (Olympus CH; Olympus, Tokyo, Japan); camera (Leica DM 750).

### Preparation of polyphenol extract of *Ocimum gratissimum* leaves

The *Ocimum gratissimum* leaves were collected from a garden at Usi, Ekiti state, Nigeria. Relevant institutional permissions to collect *Ocimum gratissimum* were obtained. The present study complies with the international, national and/or institutional guidelines for the use of different parts of the plant. The specimens of the plant were authenticated by Mr. Omole, the curator at the herbarium of Botany Department, Obafemi Awolowo University, Ile-Ife, where a voucher specimen (1945) was deposited in the Herbarium of the University. The polyphenol rich extract was obtained from the *Ocimum G.* leaves (900 g) according to our previous extraction methods of the plant [14]. Methanol was used to obtained the final extraction product, polyphenol rich extract of *Ocimum gratissimum* (PREOG) leaves under reduced pressure at 40 °C using a rotary evaporator. The resulting extract concentrate was freeze dried with the aid of a lyophilizer. The residue (80.85 g) was kept in petri dishes with a tight fitting cover and stored at − 20 °C until it was needed for the study.

$${\displaystyle \begin{array}{c}\mathrm{Extraction}\ \mathrm{yield}\ \mathrm{in}\%=80.85/900\ast 100\\ {}=8.98\%.\end{array}}$$

The OG extract obtained was dissolved in water (0.2 mL/administration) and given to rat according to the designed dosage.

### Gas chromatography–mass spectrometry analysis of (GC-MS) OG extracts

The chemical composition of the extract obtained was checked using gas chromatography–mass spectrometry (GC–MS). Kavaz et al. [34] method was adopted with slight modification. A fused-silica capillary column (film thickness of 30 × 0.25 HP-5Ms, 0.25 μm) containing helium as the carrier gas (1/mL flow rate) was used for the determination of the compounds. 40 °C oven temperature was set and held for 5 min, and then augmented gradually by 3 °C/min up to 270 °C. 60:1 was set as split ratio. Temperature of 180 °C was set for the connection parts and ion sources, and an interface temperature of 240 °C was set for the mass spectrometer. For the ionization energy quantity, 70 eV was used, while the electron impact (EI) mode was chosen to produce stable and reproducible mass spectra and a value range of 50–650 m/z was used for the running of samples. The MS delay time before scanning was 5 min. The compounds obtained was displayed in Table 2.

### Determination of total phenolic content

The total phenolic content (TPC) of the extracts was evaluated using the Folin–Ciocâlteu technique as described by Gulcin et al. [35], with a few modifications. Briefly, 100 μL (0.5 mg/mL) of the polyphenol rich extracts of *Ocimum G.* was placed into separate plain tubes. About 500 μL (1% v/v) of Folin–Ciocâlteu phenol reagent was added to each tube containing the *Ocimum G.* extract and the tubes were allowed to stand for 5 min. and then vortexed. After allowing stand for 5 min., about 400 μL (20% w/w) of sodium carbonate was added to the aliquots, which were then incubated in the dark at room temperature for 90 min. For the color development. The absorbance of each mixture was evaluated with an ultraviolet–visible spectrophotometer (Beckman, DU 7400, USA) at 765 nm. Sodium carbonate solution without any addition of Folin–Ciocâlteu phenol reagent was used as a blank. For the calibration curve, gallic acid standards were used. TPCs of the samples were determined from the linear regression of the gallic acid standards. The results were represented as the gallic acid equivalent (GAE) per gram of dry weight of *Ocimum gratissimum* extract (mg GAE/g). The procedure was conducted in triplicate (*n* = 3).

### DPPH free radical scavenging assay

The free radical scavenging activity of the extract was determined according to the method described by Rakmai et al. [36], with slight modifications. About 2 mL of DPPH– methanol solution (180 μmol/L) was added and mixed with the extract at 0.1 mg/mL. The aliquots were incubated in the dark at 25 °C. The absorbance of the sample was determined using a spectrophotometer at 517 nm at different time intervals between 0 and 60 min. Aliquots of the extracts without addition of DPPH–methanol solution were used as blanks. Trolox, a synthetic analog of vitamin E, was used as a positive control. Equation below was used to evaluate the scavenging properties of the extracts. The procedure was carried out in triplicate (*n* = 3).

$$\%{\mathrm{DPPH}}_{\mathrm{scavenging}}\kern0.5em =\kern0.5em \frac{\left[\left({\mathrm{A}}_{\mathrm{extract}}-{\mathrm{A}}_{\mathrm{blank}}\right)\kern0.5em \mathrm{x}\kern0.5em 100\right]}{{\mathrm{A}}_{\mathrm{control}}}$$

Where A_sample_ is the extract + DPPH, A_blank_ is the extract only, A_control_ is the absorbance of the control solution (containing only DPPH).

### Animals care

Twenty eight (28) male Wistar rats weighing 120-180 g were used for the study. They were procured from the Animal Holding of the College of Health Sciences, Obafemi Awolowo University, Ile-Ife. All the rats were kept in convectional cages for 1 week to acclimatize to the environmental condition at 28-32 °C and they were allowed have free access to diet and clean drinking water. The experiment was approved by the Health Research Ethics Committee (HREC) of Obafemi Awolowo University and the study has been described in accordance with the ARRIVE guidelines (animals in research: reporting in vivo experiment) [37].

### Experimental design

The animals were divided into 4 groups of 7 rats each.

- Group I: the control rats were orally treated with 2 mL/kg of water once daily for 7 consecutive days.
- Group II: rats were given 400 mg/kg body weight oral dose of PREOG for 7 consecutive days [14].
- Group III (CP): rats were maintained on a rat chow for the first 4 days before injected with CP (50 mg/kg body weight, intraperitoneally [i.p.]) for the remaining 3 days of the study [11].
- Group IV: rats were first pretreated with oral dose of PREOG at 400 mg/kg body weight for 4 consecutive days following intraperitoneal injection of CP at 50 mg/kg along with PREOG for the remaining 3 days of the study.

### Measurement of urine, food consumption and body weight

The urine of each rat in the groups was collected before and 24 h after last day of the drugs and extract of *Ocimum G.* administration for biochemical assay using metabolic cages while the food consumption and body weight of each experimental rat was measured at the beginning and the end of the experiment by digital weighing balance (Hanson, China).

### Biochemical analyses

Twenty-four hours after the last treatment, the animals were sacrificed under ketamine hydrochloride anesthesia (25 mg/kg/b.w via intramuscular route). Blood was collected from each animals by cardiac puncture and placed into separate lithium heparinized tubes. Blood obtained was centrifuged at 4000×g for 15 min at 4 °C to separate the plasma. The plasma was collected into separate plain tubes for the assessment of renal functions. The left kidney of each rat was excised and homogenized in 50 mM Tris–HCl buffer (pH 7.4) containing 1.15% potassium chloride, and the homogenate was centrifuged at 3000*×g* for 15 min. at 4 °C. The supernatant was collected for the assessment of superoxide dismutase (SOD), which was assayed by the method described by Misra and Fridovich [38]. Catalase (CAT) activity that was estimated using hydrogen peroxide as substrate according to the method of Aebi [39]. Reduced glutathione (GSH), which was determined using the method described by Beutler [40]. In addition, the hydrogen peroxide was determined from the homogenate by the method of Wolff [41] while lipid peroxidation was measured as malondialdehyde (MDA) according to the method described by Ohkawa et al. [42] and expressed as micromoles of MDA per gram tissue. Myeloperoxidase (MPO) as marker of inflammation and oxidative stress was measured according to the method of Xia and Zweier [43]. The absorbance was read at 350 nm. The right kidney of each rat was carefully excised, weighed and fixed in 10% buffer formalin for histopathological studies using periodic acid-Schiff (PAS) stain.

### Assays of urea, creatinine, cystatin C and albumin

Plasma creatinine, urea, and albumin levels were determined by biochemical kits purchased from Randox Laboratories (Crumlin, Co. Antrim, UK). The urine concentrations of the aforementioned parameters were estimated using procedures from Randox kit.

Creatinine clearance (CCr) was subsequently calculated using the standard conventional formula as a measure of glomerular filtration rate (GFR).

$$\mathrm{CCr}\kern0.5em =\kern0.5em \frac{{\mathrm{U}}_{\mathrm{Cr}}\times {\mathrm{V}}_{\mathrm{U}}}{{\mathrm{P}}_{\mathrm{Cr}}}$$

where U_Cr_ is urine creatinine concentration in μmol/L, V_U_ is urine flow rate (volume of urine/time, in mL and 24 h (1440 min), respectively), and P_Cr_ is plasma creatinine concentration in μmol/L. Plasma and urine cystatin C were determined using specific rat ELISA kits purchased from Abcam (USA).

### Plasma and urine concentration of electrolytes

The plasma and urine concentrations of sodium (Na^+^), chloride (Cl^−^) and potassium (K^+^) ions were measured. Na^+^ and K^+^ were measured by flame photometry using PFP7 (Jenway) flame photometer. Cl^−^ was assessed by using Teco laboratory kit.

### Estimations of plasma C-reactive protein (CRP), interleukin-6 and aldosterone

Plasma CRP and aldosterone were determined using specific rat ELISA kits purchased from Abcam (USA). Plasma (IL)–6 assay was estimated according to the manufacturer’s instructions using the ELISA kits obtained from Elabscience (Texas, USA).

### Histopathological studies

Each rat right kidney was fixed in 10% formo-saline. The tissues were cut into 3-4 μm thick sections by a microtome, fixed on the slides and stained with PAS. The slides were observed under a light microscope (Olympus CH; Olympus, Tokyo, Japan) and photomicrographs were taken with a Leica DM 750 camera at × 400 magnifications. The renal tissues section were assessed by a certified pathologist who was blinded to the experimental protocol. Glomerular damage was scored between grade 0 and 4 by the evaluation of mesangial proliferation, basal membrane thickness, and fibrinoid changes. Tubular damage was scored between grade 0 and 4 by the evaluation of vacuolar degeneration, desquamation, tubular degeneration, proximal tubular necrosis, and cast formation. Tubulointerstitial inflammatory infiltrates were scored between grade 0 and 2 by the evaluation of diversity of inflammatory cells in the medium and the presence of infiltrates within tubules epithelium (Table 1) [44]. Total renal damage score of 0–2 represented none/mild nephrotoxicity, 3–6 moderate nephrotoxicity, and 7–10 severe nephrotoxicity [44].

**Table 1:** Nephrotoxicity scoring system in histopathology

Grade	Glomerular Damage	Tubular damage	Tubulointerstitial inflammatory infiltrates
0	None (Normal)	None (Normal)	None (Normal)
1	< 25% of Glomeruli	< 25% of parenchymal tubules	Leucocytes only in interstitium
2	25–50% of Glomeruli	25–50% of parenchymal tubules	Leucocytes in both interstitium and tubular epithelial cells
3	50–75% of Glomeruli	50–75% of parenchymal tubules	_
4	> 75% of Glomeruli	> 75% of parenchymal tubules	_

Total score of 0–2 represents none/mild nephrotoxicity, 3–6 moderate nephrotoxicity, 7–10 severe nephrotoxicity

### Statistical analysis

All data were expressed as means ± standard errors of means (S.E.M). The statistical analysis was performed using one-way analysis of variance (ANOVA) followed by Neumann Keul’s post hoc test for comparison between groups. Differences were considered significant when *p* < 0.05 (Graph Pad Software Inc., San Diego, CA, USA).

## Results

### Gas chromatography–mass spectrometry analysis, phenolic contents and DPPH scavenging capacity of the *Ocimum gratissimum* leaves extract

GC–MS analysis revealed some bioactive compounds of *Ocimum gratissimum* leaves extract (Table 2). The polyphenolic contents of the extract was recognized by using the Folin–Ciocâlteu method (Table 2). The Total phenolic contents (TPC) (80.36 ± 0.50 mg GAE/100 g) obtained in *Ocimum G.* extract revealed that *Ocimum gratissimum* extract are rich in phenolic contents.

**Table 2:** Chemical structure, phenolic contents and DPPH scavenging capacity of the *Ocimum gratissimum* leaves extract

**Retention time (min)**	**Chemical name**	**Chemical Structure**	**Content (%, wt/wt) OG**
6.0010	Methyl ester	CH_3_ – RCOOR	0.039
6.0710	Glycerin	C_3_H_8_O_3_	0.56
17.062	*p*-Cymene	C_10_H_14_	5.52
20.402	Sabinene	C_10_H_16_	4.61
42.395	2-isopropyl-5-methylphenol (Thymol)	C_10_H_14_O	0.20
45.311	α-copaene	C_15_H_24_	0.96
47.012	Trans-caryophyllene	C_15_H_24_	0.40
50.005	β-Selinene	C_15_H_24_	0.31
52.102	Phenol, 3-(1,1-dimethylethyl)-4-methoxy-	C_12_H_18_O_2_	0.90
54.633	Caryophyllene oxide	C_15_H_24_O	2.07
64.065	Neophytadiene	C_20_H_38_	2.99
65.100	Phytol	C_20_H_40_O	0.63
70.355	Carvacrol (2-methyl-5-(propan-2-yl)phenol	C_10_H_14_O	0.92
**Total Phenolic Content of the Extract**			
**EXTRACT**		**TPC (mg GAE/100 g)**	
*Ocimum gratissimum* leaves		80.36 ± 0.50	
**DPPH Scavenging Capacity of the Extract**			
**EXTRACT**		**Scavenging activity (IC**_**50**_**; mg/mL)**	
*Ocimum gratissimum* leaves		22.12 ± 0.43	

Half-maximal concentration (IC_50_) values for the DPPH free radical-scavenging activity assay and content of phenolic of *Ocimum gratissimum* extract. Values shown are means of three replicates (*n* = 3) ± SEM

The DPPH scavenging capacity of the extract was also evaluated by calculating the IC_50_ value, which relates to the amount of extract that is capable of scavenging 50% of the free radicals contained in the reaction mixture. The IC_50_ (22.12 ± 0.43 mg/ml) value of the extract was high and this denoted high antioxidant activity of *Ocimum gratissimum* leaves. Thus the function of the plant high phenolic content was shown in its ability to scavenge free radical activity (Table 2).

### Effect of PREOG and cyclophosphamide on water consumption (ml), urine output (ml), food consumption and body weight (g) of the rats

The water consumption and urine output of rats treated with CP only were significantly higher (*p* < 0.05) when compared with the control and PREOG + CP groups. However, the rats in PREOG + CP group had no significant difference (*p* > 0.05) in water consumption and urine output when compared with the control (Table 3).

**Table 3:** Effects of polyphenol rich extract of *Ocimum gratissimum* leaves on water intake, urine output, food consumption (g) and body weight (g) in cyclophosphamide-treated rats

	Baseline	24 h. after 7 days of administration of drugs
**Water Intake (ml/24 h)**		
[1] Control	17.70 ± 0.40	18.70 ± 0.59
[2] PREOG 400 mg/kg	15.70 ± 0.73	19.60 ± 0.43
[3] CP (50 mg/kg)	18.30 ± 1.69	30.80 ± 1.33*
[4] PREOG 400 mg/kg + CP	15.90 ± 1.76	20.10 ± 1.66^#^
**Urine Output (ml/24 h.)**		
[1] Control	12.60 ± 0.21	13.10 ± 0.19
[2] PREOG (400 mg/kg)	13.10 ± 0.35	14.60 ± 0.33
[3] CP (50 mg/kg)	14.00 ± 1.39	20.85 ± 1.03*
[4] PREOG (400 mg/kg) + CP	14.11 ± 1.44	16.00 ± 1.46^#^
**Food consumption (g)**		
[1] Control	11.60 ± 0.80	15.80 ± 0.13
[2] PREOG (400 mg/kg)	14.80 ± 0.43	20.60 ± 0.33
[3] CP (50 mg/kg)	12.80 ± 1.29	10.06 ± 1.03*
[4] PREOG (400 mg/kg) + CP	12.20 ± 1.42	14.50 ± 1.06^#^
**Body weight (g)**		
[1] Control	135.40 ± 3.37	150.00 ± 2.44
[2] PREOG (400 mg/kg)	150.00 ± 1.75	170.20 ± 4.00
[3] CP (50 mg/kg)	145.20 ± 4.89	129.10 ± 2.01*
[4] PREOG (400 mg/kg) + CP	150.60 ± 6.47	146.40 ± 3.16^#^

Each value represents mean ± SEM (*n* = 7). * (*p* < 0.05) = significantly different from control in the same column. ^#^ (*p* < 0.05) = significantly different from CP (50 mg/kg) in the same column

The group treated with CP only had lowered food intake and body weight when compared with the control and PREOG + CP groups. However, the rats in PREOG + CP group exhibited no significant difference (*p* > 0.05) in food intake and body weight when compared with the control (Table 3).

### Effect of PREOG and cyclophosphamide on some parameter of renal of functions

The plasma creatinine, urea, cystatin C and albumin of CP treated group were significantly elevated in comparison to the control (*p* < 0.05) (Table 4). However, the urinary output of these parameters (creatinine, urea, cystatin C and albumin) remained significantly reduced when compared with the control. The PREOG group had no significant difference (*p* > 0.05) in the plasma and urine levels of creatinine, urea, cystatin C and albumin, except the plasma urea that showed elevated level when compared with control (Table 4). Thus, the polyphenol rich extract of *Ocimum gratissimum* leaves prevented the adverse effect of CP on the aforementioned parameters (Table 4).

**Table 4:** Effects of polyphenol rich extract of *Ocimum gratissimum* leaves on some parameter of renal function in cyclophosphamide-treated rats

**Creatinine Concentration**	**Plasma Level**	**Urine Level**
**Groups**	**(μmol/L)**	**(μmol/L)**
[1] Control	34.48 ± 4.33	1768.4 ± 5.56
[2] PREOG (400 mg/kg)	39.79 ± 2.38	1945.24 ± 8.65
[3] CP (50 mg/kg)	168.00 ± 3.23*	970.85 ± 4.33*
[4] PREOG (400 mg/kg) + CP	44.21 ± 4.57^#^	1670.25 ± 6.45^#^
**Urea Concentration**	**Plasma Level**	**Urine Level**
**Groups**	**(mmol/L)**	**(mmol/L)**
[1] Control	5.90 ± 0.27	200.02 ± 21.39
[2] PREOG (400 mg/kg)	7.00 ± 0.92	200.90 ± 15.88
[3] CP (50 mg/kg)	16.70 ± 5.26*	130.03 ± 20.19*
[4] PREOG (400 mg/kg) + CP	11.95 ± 0.42*^#^	179.67 ± 30.38^#^
**Cystatin C Concentration**	**Plasma Level**	**Urine Level**
**Groups**	**(ng/ml)**	**(ng/ml)**
[1] Control	0.23 ± 0.31	12.34 ± 3.01
[2] PREOG (400 mg/kg)	0.25 ± 0.44	13.30 ± 2.08
[3] CP (50 mg/kg)	0.59 ± 0.66*	25.34 ± 1.19*
[4] PREOG (400 mg/kg) + CP	0.43 ± 0.23^#^	15.08 ± 1.38^#^
**Albumin**	**Plasma Level**	**Urine Level**
**Groups**	**(g/dl)**	**(mg/dl)**
[1] Control	6.44 ± 0.55	0.19 ± 0.71
[2] PREOG (400 mg/kg)	7.09 ± 0.21	0.22 ± 1.01
[3] CP (50 mg/kg)	3.22 ± 0.25*	1.25 ± 0.91*
[4] PREOG (400 mg/kg) + CP	5.78 ± 1.01^#^	0.28 ± 0.08^#^
**Albumin/Creatinine Ratio**	**(mg/g)**	
**Groups**		
[1] Control	9.91 ± 0.51	
[2] PREOG (400 mg/kg)	10.70 ± 1.32	
[3] CP (50 mg/kg)	113.8 ± 4.66*	
[4] PREOG (400 mg/kg) + CP	14.80 ± 0.52^#^	

Each value represents mean ± SEM (*n* = 7). * (*p* < 0.05) = significantly different from control in the same column. ^#^ (*p* < 0.05) = significantly different from CP (50 mg/kg) in the same column

Albumin - to – creatinine ratio of CP group was significantly higher (*p* < 0.05) when compared with control and PREOG groups. The PREOG group exhibited no significant difference (*p* > 0.05) in Albumin - to – creatinine ratio when compared with the control (Table 4).

### Effect of PREOG and cyclophosphamide on creatinine clearance

**Fig. 1:**
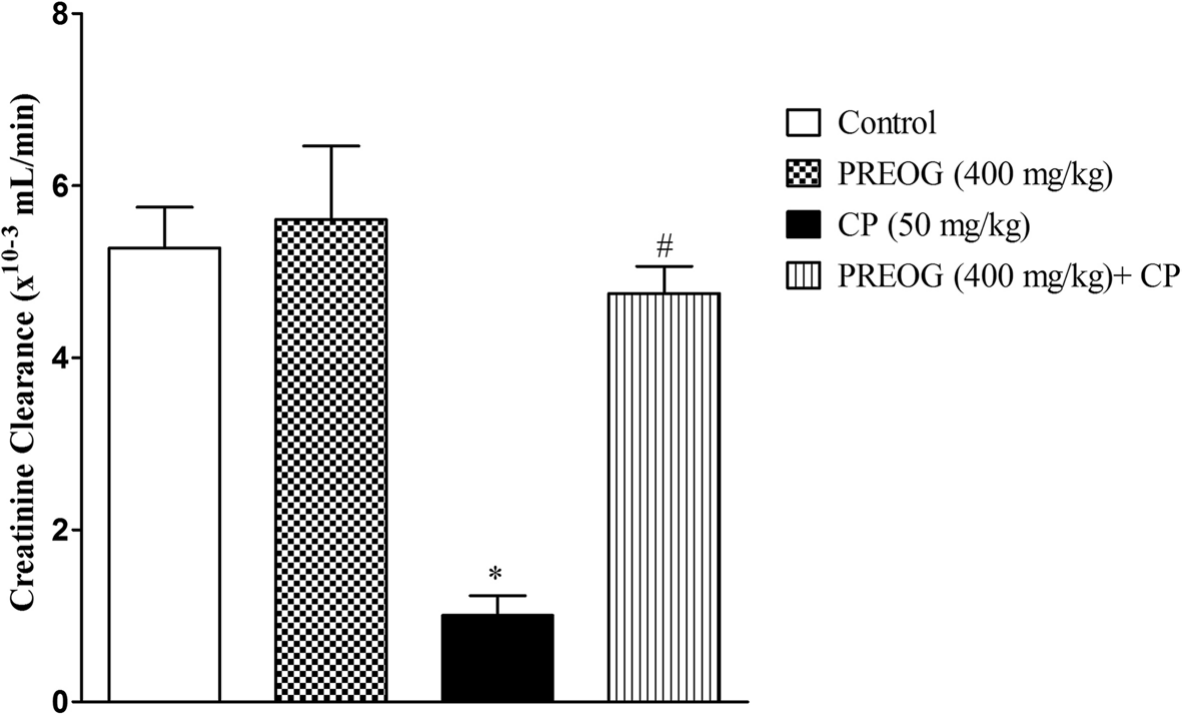
Effects of Polyphenol Rich Extract of *Ocimum gratissimum* Leaves on Creatinine Clearance in Cyclophosphamide-Treated Rats. Each value represents mean ± SEM (*n* = 7). * (*p* < 0.05) = significantly different from control. ^#^ (*p* < 0.05) = significantly different from CP (50 mg/kg)

A significant decrease in the renal creatinine clearance was observed in the CP alone group when compared with the control (*p* < 0.05). However, the PREOG-treated groups revealed a significant increase in the creatinine clearance when compared with CP alone treated group (*p* < 0.05) (Fig. 1).

### Effect of PREOG and cyclophosphamide on plasma and urine electrolytes

Treatment of rats with CP alone showed a significantly lower in plasma Na^+^ and Cl^−^ concentrations, but a significantly higher in plasma K^+^ concentration compared with the control group. The urine concentrations of Na^+^ and Cl^−^ were significantly higher in the CP alone group when compared with control and PREOG + CP groups. However, the urine excretion of K^+^ was significantly lower when compared with the control and PREOG + CP. On the other hand, treatment with PROEOG significantly alleviated the aforementioned parameters (Table 5).

**Table 5:** Effects of polyphenol rich extract of *Ocimum gratissimum* leaves on plasma and urine electrolytes in cyclophosphamide-treated rats

Groups	Plasma Electrolytes			Urine Electrolytes		
	Na^**+**^	K^**+**^	Cl^**−**^	Na^**+**^	K^**+**^	Cl^**−**^
	(mmol/L)	(mmol/L)	(mmol/L)	(mmol/L)	(mmol/L)	(mmol/L)
**[1] Control**	149.80 ± 2.58	5.08 ± 0.12	112.20 ± 3.49	155.0 ± 5.53	134.87 ± 6.38	130.00 ± 2.45
**[2] PREOG**	152.70 ± 2.80	5.12 ± 0.10	118.80 ± 4.01	158.00 ± 5.00	140.03 ± 3.91	138.67 ± 1.38
**[3] CP**	131.00 ± 5.10*	6.96 ± 0.13*	96.20 ± 6.76*	202.00 ± 9.13*	98.00 ± 2.05*	192.00 ± 5.67*
**[4] PREOG + CP**	142.90 ± 2.06^#^	5.45 ± 0.09^#^	108.20 ± 2.99^#^	166.00 ± 5.07^#^	129.89 ± 1.24^#^	140.10 ± 6.09^#^

Each value represents mean ± SEM (*n* = 7). * (*p* < 0.05) = significantly different from control in the same column. ^#^ (*p* < 0.05) = significantly different from CP (50 mg/kg) in the same column

### Effect of PREOG and cyclophosphamide on aldosterone

**Fig. 2:**
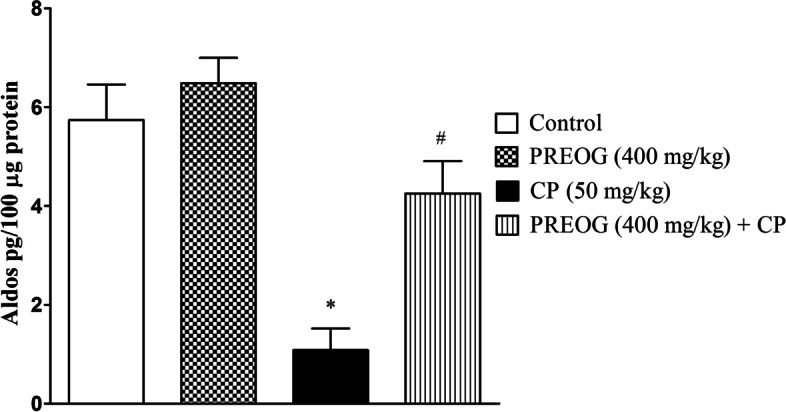
Effects of Polyphenol Rich Extract of *Ocimum gratissimum* Leaves on Aldosterone in Cyclophosphamide-Treated Rats. Each value represents mean ± SEM (*n* = 7). * (*p* < 0.05) = significantly different from control. ^#^ (*p* < 0.05) = significantly different from CP (50 mg/kg)

CP treated group exhibited lower aldosterone level when compared with the control (*p* < 0.05). However, the PREOG-treated groups had significantly higher aldosterone level in comparison to CP alone treated group (*p* < 0.05) (Fig. 2).

### Effect of PREOG and cyclophosphamide on interleukin-6 and C - reactive protein

**Fig. 3:**
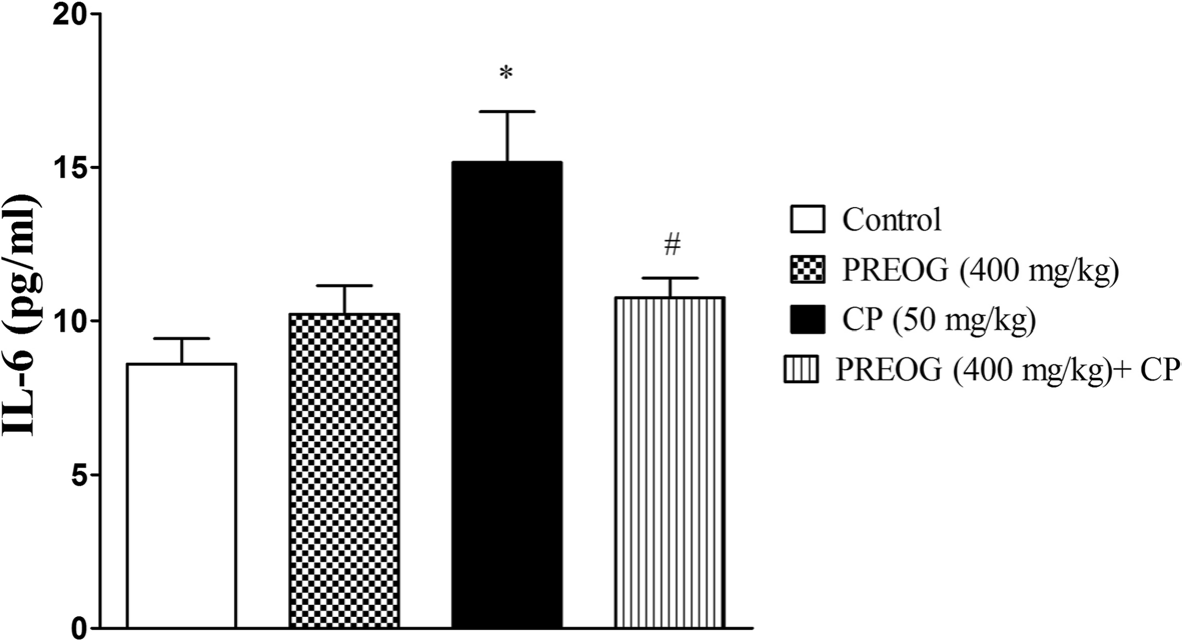
Effects of Polyphenol Rich Extract of *Ocimum gratissimum* Leaves on Interleukin-6 in Cyclophosphamide-Treated Rats. Each value represents mean ± SEM (*n* = 7). * (*p* < 0.05) = significantly different from control. ^#^ (*p* < 0.05) = significantly different from CP (50 mg/kg)

**Fig. 4:**
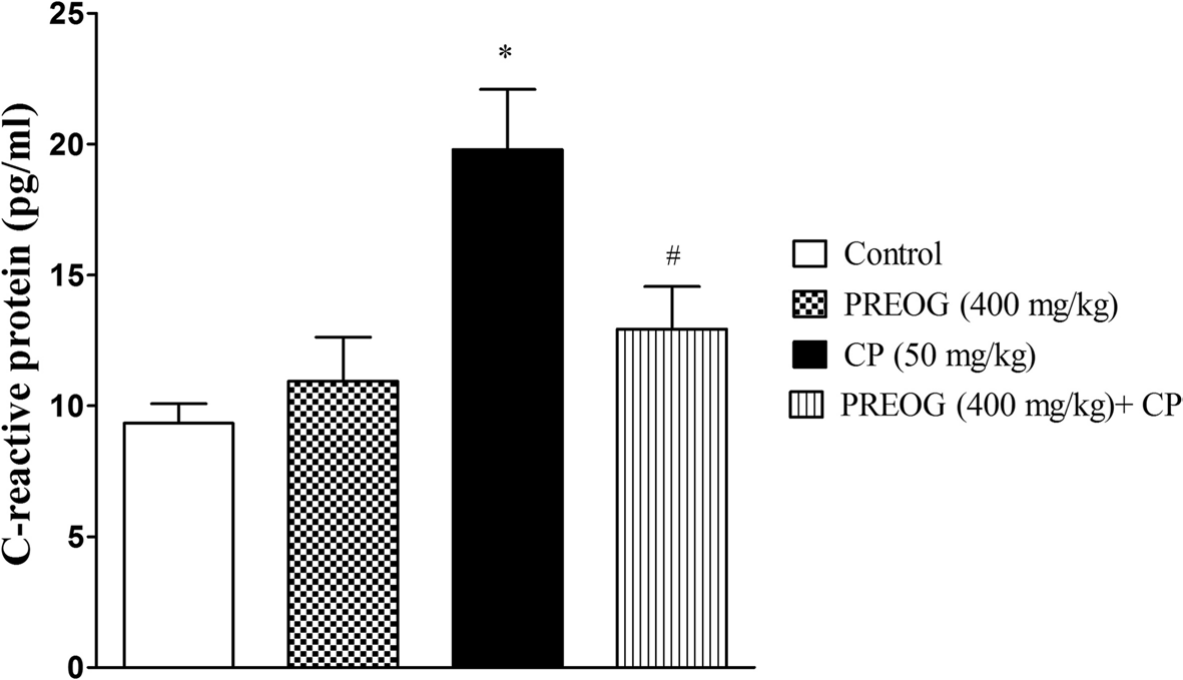
Effects of Polyphenol Rich Extract of *Ocimum gratissimum* Leaves on C-reactive protein in Cyclophosphamide-Treated Rats. Each value represents mean ± SEM (*n* = 7). * (*p* < 0.05) = significantly different from control. ^#^ (*p* < 0.05) = significantly different from CP (50 mg/kg)

Figures 3 and 4 show the effect of PREOG and CP on markers of inflammation (IL-6 and C - reactive protein) in the plasma. There was a significantly increased levels of interleukin-6 and C-reactive protein in the plasma of rats treated with CP alone when compared with control (*p* < 0.05). Polyphenol extract of *Ocimum gratissimum* leaves significantly decreased the elevated plasma IL-6 and C-reactive protein levels compared to CP-treated group (*p* < 0.05).

### PREOG repressed oxidative stress and improved antioxidant enzyme activities

Table 6 shows the effect of PREOG on the kidney antioxidant enzymes (SOD and CAT) activities, GSH, MDA and H_2_O_2_ levels. The present findings show that CP significantly decreased (*p* < 0.05) renal activities of SOD, and CAT, along with GSH level, whereas MDA and H_2_O_2_ levels were significantly increased (*p* < 0.05) compared to the control. In contrast, PREOG significantly restored the renal SOD, and CAT activities, as well as GSH, MDA and H_2_O_2_ levels to normalcy. The observed alleviation of oxidative stress in PREOG + CP treated group indicate free radicals scavenging ability of the plant extract against CP-induced renotoxicity.

**Table 6:** Effects of polyphenol rich extract of *Ocimum gratissimum* leaves on oxidative stress and cellular infiltration indices in cyclophosphamide-treated rats

Groups	Kidney SOD (μ/mg protein)	Kidney CAT (μM/mg protein)	Kidney GSH (μg/mg protein)	Kidney MDA (nM/mg protein)	Kidney H_2_O_2_ (nM/g protein)	Kidney MPO (U/mg protein)
[1] Control	2.35 ± 0.27	5.01 ± 0.35	10.04. ± 0.21	14.54 ± 0.46	24.00 ± 1.31	6.94 ± 4.05
[2] PREOG (400 mg/kg)	2.40 ± 0.03	6.08 ± 1.10	11.43 ± 0.30	16.02 ± 2.37	24.89 ± 2.30	7.09 ± 4.21
[3] CP (50 mg/kg)	1.12 ± 1.14*	2.01 ± 0.30*	5.40 ± 1.45*	30.90 ± 1.62*	40.84 ± 3.13*	35.02 ± 6.06*
[4] PREOG (400 mg/kg) + CP	2.00 ± 0.20^#^	4.01 ± 0.15^#^	9.43 ± 1.56^#^	18.31 ± 1.64^#^	29.09 ± 4.06^#^	16.08 ± 3.31*^#^

Each value represents mean ± SEM (*n* = 7). * (*p* < 0.05) = significantly different from control in the same column. ^#^ (*p* < 0.05) = significantly different from CP (50 mg/kg) in the same column

Myeloperoxidase activity was significantly higher in CP alone treated group when compared with the control. However, PREOG significantly decreased the elevated MPO activity compared to the CP treated group (Table 6).

### PREOG improves histopathological alterations in the kidney tissue

**Fig. 5:**
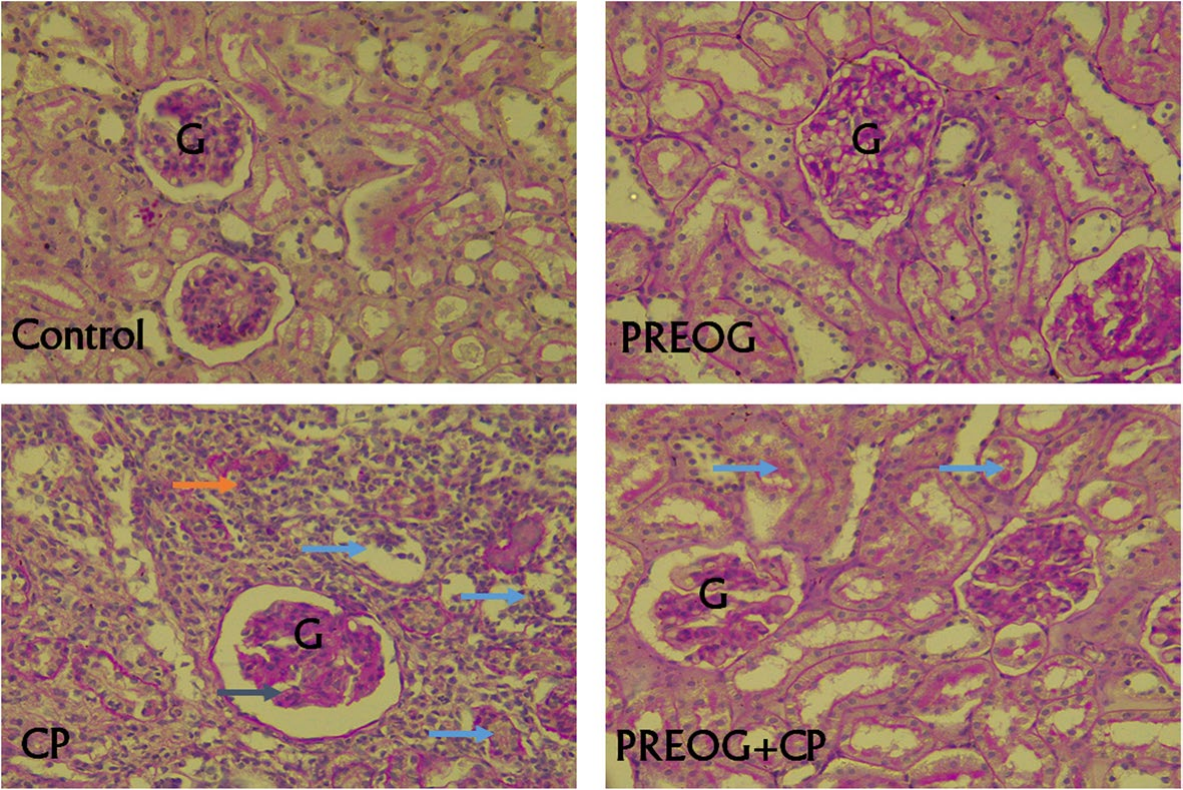
The histoarchitecture of the control, and PREOG groups show normal renal parenchymal tissue with intact glomeruli, proximal and distal convoluted tubules and loop of Henle. The blood vessels are intact. Histoarchitecture of CP shows renal tissue with marked alteration of the renal parenchymal tissue owing to severe destruction/damage of the glomeruli (black arrow), proximal and distal convoluted tubules (blue arrows). Infiltration of the interstitium by chronic inflammatory cells mostly neutrophils were also seen in CP treated rats (brown arrow). However, PREOG + CP group renal architecture show mild alteration in glomeruli and mild infiltration of the interstitium with inflammatory cells. The proximal and distal convoluted tubules and loop of Henle were intact in this group than CP alone group (PAS, X400)

The histoarchitecture of the control, and PREOG groups showed normal glomeruli and tubules as well intact blood vessels (Fig. 5). Histoarchitecture of CP showed marked alteration of glomeruli and proximal and distal convoluted tubules damage. Infiltration of the interstitium by chronic inflammatory cells mostly neutrophils were also seen in CP treated rats. However, PREOG + CP group showed mild alteration in glomeruli and mild infiltration of the interstitium with inflammatory cells. The proximal and distal convoluted tubules and loop of Henle were intact in this group than CP alone group (Fig. 5). The total nephrotoxicity score of CP alone was significantly higher than that the control and PREOG + CP groups (*p* < 0.05) (Table 7).

**Table 7:** Histopathological nephrotoxicity scores of the experimental groups

Group	Glomerular damage						Tubular damage						Tubulointerstitial inflammatory infiltrate				Total nephrotoxicity score	Average
	G0	G1	G2	G3	G4	Average	G0	G1	G2	G3	G4	Average	G0	G1	G2	Average		
1. Control	7	0	0	0	0	0.0	7	0	0	0	0	0.0	7	0	0	0.0	0	0.0
2. PREOG	7	0	0	0	0	0.0	7	0	0	0	0	0.0	7	0	0	0.0	0	0.0
3. CP	4	0	1	1	1	0.60*	3	0	1	1	2	1.40*	5	0	2	0.48*	7	0.76*
4. PREOG + CP	6	1	0	0	0	0.20^#^	6	1	0	0	0	0.20^#^	6	1	0	0.20^#^	2	0.12^#^

Total score of 0–2 represents none/mild nephrotoxicity, 3–6 moderate nephrotoxicity, 7–10 severe nephrotoxicity

*G* Grade

Each value represents mean ± SEM (*n* = 7). * (*p* < 0.05) = significantly different from control. ^#^ (*p* < 0.05) = significantly different from CP (50 mg/kg)

## Discussion

In the current study, there are indications of elevated levels in the parameters of oxidative stress such as lipid peroxidation, hydrogen peroxide (H_2_O_2_), and nitric oxide (NO), suggesting that the membrane lipids and protein of the kidney of the rats treated with CP alone were damaged. Also, the activities of antioxidant enzymes such as superoxide dismutase and catalase and level of non-enzymatic antioxidant, reduced glutathione were decreased in the group treated with CP alone. The decrease in antioxidants in the renal cells of the rats treated with CP alone was an indication of redox-homeostasis imbalance. CP has been previously shown to induce oxidative damage, inflammation, and cell necrosis or apoptosis after activation and promotion of reactive oxygen species (ROS) via its metabolite, acrolein [7, 11, 14], which subsequently trigger the deactivation and diminishing of antioxidant defense capacities of the affected organisms [10, 11, 45]. In this study, the overproduction of ROS outweighs the endogenous antioxidant capacity, triggers oxidative stress, and subsequently damages renal cells of the CP-treated rats as was reflected by the increased levels of markers of oxidative stress and decreased levels of antioxidant parameters. Also, the histology view of these rats showed glomerular damage and tubular necrosis of the renal cells.

Furthermore, redox imbalance elicits proinflammatory cytokines overproduction [3, 14]. The observed overproduction of ROS triggered lipid peroxidation and mobilization of proinflammatory cytokines and concurrently exerted inflammatory renal cell damage. IL-6 assessment in this study was significantly elevated in CP-treated rats, which indicates oxidative stress attack and inflammation of the renal cells. IL-6 elevation has been found to trigger and/or stimulate the liver to initiate acute phase response that results in an increase in circulating C-reactive protein (CRP) production and other bioactive mediators [46]. CRP was significantly increased in CP-treated rats and the elevated level of this biomarker was an indication of an early defense system against infections, inflammation, and tissue damage [47, 48]. The elevation of the CRP level in this study further confirmed undergoing inflammation in the rats treated with CP alone. CP has been observed to trigger the reduction of the kidney’s endogenous antioxidants such as overutilization of GSH by the kidney due to accumulation of cyclophosphamide metabolite products in the renal tubular cells [9]. As a result of this process, the kidney of rats treated with CP alone could upsurge immune response against its cells, which subsequently resulted in inflammation as shown by the elevation of the plasma CRP. In agreement, CP has the potential of inducing inflammation and likely immunological damage in the kidney of CP-treated rats.

GC – MS analysis confirmed some vital bioactive components of *Ocimum G.* extract and Folin–Ciocâlteu routine revealed high phenolic contents of the extract. The observed polyphenol-rich of the plant increases its antioxidant capacity against the free radicals as was revealed by the DPPH scavenging capacity of the *Ocimum G.* extract. This proved the antioxidative and anti-inflammatory effect of PREOG in vitro.

Co-administration of PREOG and CP show promising therapeutic advantages over CP treated group alone on the kidney tissue. The increase in antioxidant expression levels (SOD, CAT, and GSH) and decrease in oxidative stress markers (lipid peroxidation, H_2_O_2,_ and NO) seen in *Ocimum G.* extract groups indicated that *Ocimum G.* extracts improved antioxidant capacities and prevented renal oxidative damage via suppressing the overproduction of ROS in rats being exposed to CP toxicity. These indications proved the antioxidant and anti-inflammatory capacity of PREOG in vivo. This study is in line with the previous studies that reported residual antioxidative, anti-inflammatory, and antibacterial activities of *Ocimum G* [14, 18, 19]..

The undergoing inflammation in the renal tissue of rats treated with CP was also confirmed by an elevated level of myeloperoxidase (MPO). MPO is a marker of leukocyte infiltration and inflammatory conditions [49]. The activity of this enzyme is higher when inflammation occurs in the blood and tissues and is usually used to assess the severity of inflammation [49, 50]. MPO is found in azurophilic granules of neutrophils and macrophages. The elevated activity of this enzyme is an indication of infiltration of inflammatory cells in the kidney or other organs [49, 51]. The inflammatory process was also confirmed by the histopathological study, showing neutrophil infiltration and tubular necrosis in the renal cells of CP-treated rats. Thus, induction of inflammation is the main pathological manifestation of CP organ toxicity [7]. However, in this study, the administration of polyphenol-rich extract of *Ocimum G.*, reduced MPO activity, indicating inhibition of leucocytes infiltration and inflammation in the kidney.

As a result of the overproduction of ROS and oxidative damage of the renal cells induced by CP assault, the markers of renal function test such as plasma creatinine and urea level were significantly elevated in the group treated with CP alone which indicated marked renal impairment. The significant increase in plasma creatinine and urea levels and subsequently decrease in urine output is indicative of renal dysfunction [52]. CP-induced renal impairment was associated with an excessive increase in plasma and urinary output of cystatin C levels, which is reported to be a more sensitive marker for the diagnosis of acute kidney injury over creatinine and urea [53]. The significant increase of cystatin C level in the urine further confirmed renal dysfunction and damage in the CP-treated group. The severity of CP-induced renal dysfunction was confirmed by histopathological examination, which revealed distortion of the glomeruli, proximal and distal renal tubules. However, administration of PREOG prevented the adverse side effect of CP on some markers of renal function such as plasma levels of creatinine, urea, and cystatin C and urine levels of these markers, indicating improved kidney function. These results are consistent with those reported by Ogundipe et al. [54], where the extract of *Ocimum G.* significantly inhibited the actions of ROS in the kidney of gentamicin-treated rats. ROS have been found to cause kidney function alteration, tissue necrosis and the inability of the kidney to filter or secrete urea and creatinine from the body of the animals. Interestingly, the protective effect of PREOG in the kidneys of rats imply that leave extract of *Ocimum G.* possesses some essential antioxidant compounds as reflected in GC–MS analysis, and the antioxidant activities of *Ocimum G.* might be the reason for the reduction in the ROS and other reactive by-products generated by CP toxic metabolites in the kidney tissue of rats co-treated with PREOG and CP. Indeed, the histopathological study showed evidence of improvement in the structure of the kidney of the PREOG + CP co-treated than the CP-treated group.

Albumin is one of the carrier proteins that function to maintain oncotic pressure and extracellular fluid volume. Normal kidney function prevents this protein from passing through its filtration barrier, however, damage to the kidney structure, particularly the filtration barrier of the glomeruli, allows the filtration of this protein to pass through and subsequently being detectable in the urine. The significant decrease in the plasma level and increase in the urinary output of albumin in CP-treated rats indicated kidney dysfunction, which reflects glomerular filtration barrier dysfunction/damage. Albumin-to-creatinine ratio was used to assess albuminuria or microalbuminuria (glomerular filtration function). The presence of albuminuria or microalbuminuria is an indication of glomerular filtration dysfunction and micro-and macro-vascular dysfunctions [55, 56]. Results of this study suggested that CP contributes to glomerular filtration and endothelial dysfunction. This study was in tandem with the previous observations on the effects of CP on endothelium dysfunctions [57, 58]. However, PREOG treatment showed a significant increase in the plasma and decreased urine output of albumin. Also, albumin-to-creatinine ratio assessment in the PREOG-treated group was significantly decreased compared with CP-treated rats. These indicated that PREOG improved endothelial function and glomerular filtration membrane thereby increasing glomeruli blood perfusion.

The creatinine clearance (Ccr) assessment in this study was significantly reduced in CP-treated rats, which indicates reduced perfusion of the glomeruli. The reduction in the renal glomeruli perfusion could be due to the renal vascular constriction and/or damage to the glomerular capillary endothelium and these resulted in a reduced glomerular filtration rate. The significant decrease in creatinine clearance that was observed in the CP-treated rats is an indication of alteration of the glomerular filtration function. However, Ccr of the PREOG-treated group was significantly elevated, which indicates improved renal blood flow and glomerular filtration rate. The photomicrograph of the PREOG-treated rats further confirmed the improved renal glomeruli function.

Electrolytes are essential ions in the blood for homeostasis. However, alteration in renal function due to exposure to toxic substances could result in irregularities in the body electrolytes level [52]. The plasma sodium and chloride ions in this study were significantly lowered in CP-treated rats, suggesting cases of hyponatremia and hypochloremia. Moreover, the assessment of the urinary output of these two ions showed significant increases, which indicates an alteration in renal handling of these ions. Cyclophosphamide has been known to induce excessive sodium loss through the urine by stimulating the production of antidiuretic hormone from the hypothalamus, which therefore influences a decrease in aldosterone secretion from the adrenal cortex [59, 60]. A significant reduction of aldosterone was noted in CP-treated rats in this study. The decreased aldosterone production reduced sodium ion reabsorption in the distal convoluted tubules of the kidney nephron. The increased urinary output and decreased plasma level of sodium ion that was observed in this study was due to cyclophosphamide toxic metabolite effect on posterior pituitary, which increases ADH production to enhance water retention and indirectly halted aldosterone secretion from the adrenal cortex and thereby influences large urinary sodium excretion in the rats. However, PREOG administration normalized plasma sodium level either by activating or increasing aldosterone secretion in the adrenal cortex, thereby maintaining the amount of sodium excretion in the urine of the rats co-treated with PREOG + CP compared with the CP-treated rats.

The water intake and urine excretion of CP-treated rats significantly increased than those treated with PREOG. The sodium loss in this study could be the reason for the polyuria and polydipsia that were observed in the CP-treated group. PREOG administration prevented excessive sodium and water loss through urine in rats co-treated with PREOG + CP compared with the CP-treated rats.

Plasma potassium level was significantly increased and significantly lowered in the urine of CP-treated rats. Hyperkalemia is one of the markers of renal injury because it depends on the glomerular filtration and tubular secretion for excretion [44, 61]. The excessive elevated level of plasma potassium ion that was observed is a sign of compromised renal function, particularly tubular damage, and could also be linked to cardiovascular diseases risk of CP effect. However, treatment with PREOG restored plasma potassium level and prevented CP-induced hyperkalemia in treated rats. This attribute indicates that polyphenol-rich extract of *Ocimum gratissimum* leaves have antioxidant and membrane stabilizing properties.

Cyclophosphamide treatment in this study was associated with anorexia, a significant reduction in food intake, and mild tiredness. This explains the reduction in body weight that was observed in the animals. Healthy body weight is a product of an equilibrium between food consumption and the rate of energy expenditure [44]. Hence, the bodyweight reduction and anorexia that were observed could be due to CP exposure effect on gastrointestinal disturbance [62]. However, the administration of PREOG improved food consumption and prevented the reduction in body weight and enhanced fitness of the rats. This finding is in support of Ogundipe et al. [63], who reported an increase in body weight of rats treated with extract of *Ocimum G.* after gentamicin administration. This suggests that *Ocimum G.* extract improves the energy balance between the rate of caloric consumption and expenditure in rats. These features showed the health beneficial effect of PREOG over CP toxicity in rats.

## Conclusion

In conclusion, the results from the present findings showed that polyphenol-rich extract of *Ocimum gratissimum* prevented adverse side effects of CP on the renal function via decreasing oxidative stress, prevented overproduction of pro-inflammatory cytokines and leukocytes infiltration, and improvement of antioxidant enzymatic activity of the renal tissues. Moreover, it was confirmed that CP induced reduction in aldosterone secretion, and facilitated excessive sodium and water loss through urine in the rats. However, extract of *Ocimum G.* improved renal function, increased aldosterone production, and prevented excessive sodium and water loss in CP-treated rats. The presence of polyphenol and antioxidants in the leaves of *Ocimum gratissimum* make it a perfect candidate for the treatment and/or prevention of the nephrotoxic effect of cyclophosphamide.

## Data Availability

The datasets used and/or analyzed during the current study available from the corresponding author on reasonable request.

## Abbreviations

Ocimum G.: *Ocimum gratissimum*
CP: Cyclophosphamide
PREOG: Polyphenol rich extract of *Ocimum gratissimum*
GC – MS: Gas chromatography–mass spectrometry
CRP: C-reactive protein
MPO: Myeloperoxidase
IL-6: Interleukin-6
MDA: Malondialdehyde
SOD: Superoxide dismutase
CAT: Catalase
LPO: Lipid peroxidation
GSH: Reduced glutathione
H_2_O_2_: Superoxide radical
ROS: Reactive oxygen species
PAS: Periodic acid-Schiff
